# Chemoradiation for the treatment of epidermoid anal cancer: 13-year follow-up of the first randomised UKCCCR Anal Cancer Trial (ACT I)

**DOI:** 10.1038/sj.bjc.6605605

**Published:** 2010-03-16

**Authors:** J Northover, R Glynne-Jones, D Sebag-Montefiore, R James, H Meadows, S Wan, M Jitlal, J Ledermann

**Affiliations:** 1 St Mark's Hospital, Northwick Park, Watford Road, Harrow, Middlesex HA1 3UJ, UK; 2 Mount Vernon Cancer Centre, Mount Vernon Hospital, Northwood, Rickmansworth Road, Northwood, Middlesex HA6 2RN, UK; 3 St James's Institute of Oncology, St James's University Hospital, Beckett Street, Leeds LS9 7TF, UK; 4 Kent Oncology Centre, Maidstone General Hospital, Hermitage Lane, Maidstone, Kent ME16 9QQ, UK; 5 Cancer Research UK & UCL Cancer Trials Centre, University College London, 90 Tottenham Court Road, London W1T 4TJ, UK

**Keywords:** anal cancer, chemoradiation, long-term follow-up

## Abstract

**Background:**

The first UKCCCR Anal Cancer Trial (1996) demonstrated the benefit of chemoradiation over radiotherapy (RT) alone for treating epidermoid anal cancer, and it became the standard treatment. Patients in this trial have now been followed up for a median of 13 years.

**Methods:**

A total of 577 patients were randomised to receive RT alone or combined modality therapy using 5-fluorouracil and mitomycin C. All patients were scheduled to receive 45 Gy by external beam irradiation. Patients who responded to treatment were recommended to have boost RT, with either an iridium implant or external beam irradiation. Data on relapse and deaths were obtained until October 2007.

**Results:**

Twelve years after treatment, for every 100 patients treated with chemoradiation, there are an expected 25.3 fewer patients with locoregional relapse (95% confidence interval (CI): 17.5–32.0 fewer) and 12.5 fewer anal cancer deaths (95% CI: 4.3–19.7 fewer), compared with 100 patients given RT alone. There was a 9.1% increase in non-anal cancer deaths in the first 5 years of chemoradiation (95% CI +3.6 to +14.6), which disappeared by 10 years.

**Conclusions:**

The clear benefit of chemoradiation outweighs an early excess risk of non-anal cancer deaths, and can still be seen 12 years after treatment. Only 11 patients suffered a locoregional relapse as a first event after 5 years, which may influence the choice of end points in future studies.

Epidermoid anal cancer is relatively uncommon, with ∼750 cases per year in the United Kingdom and 4750 in the United States (Cancer Research UK, 2008; Jemal *et al*, 2009). The first report, published in 1996, showed that radiotherapy (RT) plus chemotherapy (5-fluorouracil and mitomycin C) was superior to RT alone (UKCCCR Anal Cancer Trial Working Party, 1996).

Chemoradiation reduced the risk of local failure by 46% (*P*<0.0001) and the risk of death from anal cancer by 29% (*P*=0.02). As a consequence of this trial and of two other phase III studies (Flam *et al*, 1996; Bartelink *et al*, 1997), the combination of chemotherapy–radiation became the international standard treatment. The first UKCCCR Anal Cancer Trial (ACT I) was intended to be both pragmatic and nationally inclusive. Patients with anal margin cancers and T1 cancers, normally excluded from other randomised trials because of their more favourable prognosis, formed 23 and 13%, respectively, of the population within the trial.

The first ACT I results were based on a median follow-up of 3.5 years, but we continued to collect data on relapse and death.

## Materials and methods

A total of 585 patients were randomised to ACT I between December 1987 and March 1994 from 56 centres (53 in the United Kingdom and 3 in South Africa, Portugal and Italy). Details have been previously reported (UKCCCR Anal Cancer Trial Working Party, 1996).

### Patients

Eligible patients had epidermoid carcinoma (squamous, basaloid or cloacogenic) of the anal canal or margin. Patients were mainly clinically rather than radiologically staged. The tumour was staged using the 1985 UICC TNM classification (Spiessl *et al*, 1985). Staging for distant disease was carried out according to local practice.

### Treatments

Patients were randomised to RT alone or RT combined with chemotherapy, i.e., combined modality therapy (CMT), using a 1 : 1 allocation ratio. Radiotherapy consisted of a central axis dose of 45 Gy, delivered with anterior and posterior opposing fields. The dose was administered in 20 or 25 fractions over 4 or 5 weeks by external beam irradiation. Combined modality therapy had the same radiotherapy regimen combined with 5-fluorouracil (1000 mg m^−2^ for 4 days or 750 mg m^−2^ for 5 days) by continuous intravenous infusion during the first and final weeks of radiotherapy, and mitomycin C (12 mg m^−2^) as a single intravenous bolus injection on day 1 of the first cycle of chemotherapy. Patients judged to be good responders by 6 weeks after completing treatment were recommended to have further radiotherapy (boost), with either an iridium implant to 25 Gy (10 Gy per day) or external beam irradiation giving 15 Gy in six fractions.

### End points

Outcome measures were time to first locoregional relapse; relapse-free survival (RFS: time to any first relapse or death); colostomy-free survival (time to colostomy or death); overall survival (OS); death from anal cancer; and death from other causes. Colostomy-free survival was examined separately from locoregional failure. All colostomies, from disease and morbidity, were included.

Overall, 45% of deaths among those with known causes of death were not due to anal cancer; therefore, patients whose cause of death was unknown were classified as dying because of causes other than anal cancer. Fifteen patients who had metastases at entry to the trial were classified as having a distant relapse at the time of randomisation (six RT alone, nine CMT). Patients with pelvic or local relapse were grouped as having locoregional relapse.

Toxicities occurring or persisting more than 6 months after completing initial radiotherapy were defined as late morbidity. These were not scored or quantified. In 2007, we contacted all centres to request the following: the date and cause of death; the date of first relapse (local, pelvic or distant); and the date when the patient was last seen alive if they had not died. Death notifications in the United Kingdom were also received from the Office for National Statistics. The analysis reported here is based on data collected up to October 2007, and data on colostomies and late morbidity up to 2000.

### Statistical considerations

Kaplan–Meier survival curves and Cox regression were used to compare treatment groups. Hazard ratios (HRs) are expressed as CMT *vs* RT alone. The event rate in the CMT arm and absolute risk differences were calculated by applying the HR to the event rate in the RT alone group. Proportional hazards between treatments were assumed (i.e., the ratio of risks for having an event is constant over the study period). When this assumption did not hold, the difference in the event rate was estimated using the two observed rates. Statistical analyses were performed using Stata (version 10).

In the original analysis, several criteria were combined to define local failure: (i) the date of biopsy or clinical determination of residual/recurrent locoregional disease; (ii) the date of colostomy forming surgery for treatment-related morbidity; or (iii) 6 months after the end of treatment if a pretreatment colostomy had not been closed. Local failure was assessed from 6 weeks after initial treatment. In this paper, only treatment failures due to disease within the anal canal, margin or pelvic region were included.

In the analysis of time to first locoregional relapse, four patients who were recorded as having a distant relapse before a locoregional relapse were censored at the date of distant relapse. Patients who had any relapse before 6 weeks after the end of initial treatment were censored at the date of first relapse. For the analysis of RFS, patients with both a locoregional and distant relapse recorded on the same date are treated as having an event.

## Results

Of the 577 eligible patients randomised, 285 were allocated to receive RT alone and 292 CMT. Eight patients were found to be ineligible and were excluded from both previous and current analyses (seven patients did not have anal cancer and the other patient had previously undergone anorectal excision). The median follow-up was 13.1 years, representing 3685 patient-years in total (based on all patients and censoring those who had died); maximum follow-up was 18.9 years. We did not have data on 93 patients from October 2005. In the original report of ACT I, there were nine patients without any follow-up data, but now these data are available.

### Locoregional failure

**Figure 1:**
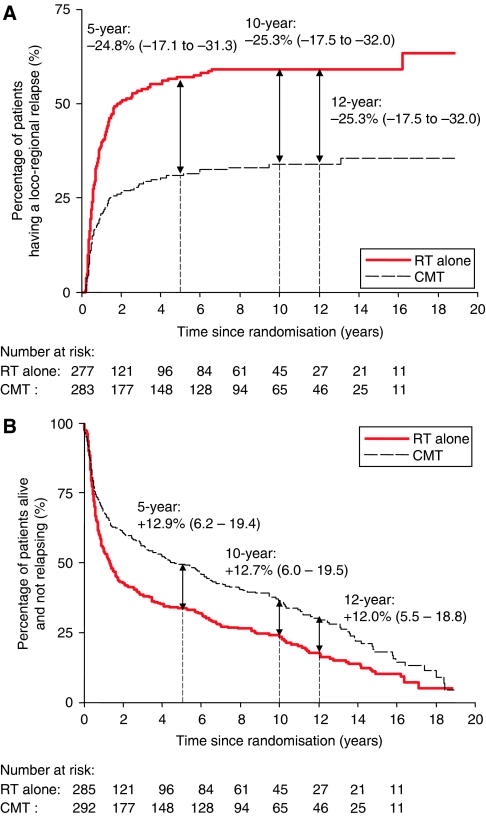
(**A**) Risk of locoregional relapse, by treatment. Estimates shown are the absolute risk differences: combined modality therapy (CMT) minus radiotherapy (RT) alone (95% confidence interval (CI)). Number of locoregional relapses: RT alone: 151; CMT: 84 (excludes deaths and relapses within 6 weeks from the end of initial treatment). Hazard ratio (HR): 0.46 (95% CI: 0.35–0.60). (**B**) Relapse-free survival, by treatment. Estimates shown are the absolute risk differences: CMT minus RT alone (95% CI). Median: RT alone: 1.3 years; CMT: 4.6 years. HR: 0.70 (95% CI: 0.58–0.84).

In all, 263 patients had locoregional relapse as their first event (Table 1). Of 560 patients, 239 had locoregional relapse (153 radiotherapy, 86 CMT), after excluding 17 (8 RT alone, 9 CMT) who did not survive 6 weeks beyond the end of initial radiotherapy (or when it was scheduled to end). Combined modality therapy was associated with a reduction in the risk of locoregional relapse, HR 0.46 (95% confidence interval (CI): 0.35–0.60), *P*<0.001). By 5 years, the absolute risk difference was −24.8% (Appendix Table A1 and Figure 1A). After 5 years, only 11 patients (five RT alone, six CMT; Appendix Table A2) developed locoregional relapse as their first event and hence the risk difference remained ∼25% thereafter.

### Relapse-free survival

A total of 452 patients died from any cause, or relapsed (Table 1). Combined modality therapy was associated with a reduction in the risk of relapsing or dying (HR 0.70, 95% CI 0.58–0.84, *P*<0.001). The absolute risk difference for death or relapse reached +12.9% at 5 years (Appendix Table A1 and Figure 1B). After 5 years, only 17 patients relapsed and 100 died; the risk difference remained fairly constant: +12% at 12 years.

Combined modality therapy was associated with a reduction in the risk of any relapse or death due to anal cancer (HR 0.61, 95% CI 0.49–0.76, *P*<0.001). The absolute risk difference remained similar after 2 years, and at 12 years was −18.0% (95% CI: −10.2 to −25.4%) in favour of CMT.

### Colostomy-free survival

**Figure A1:**
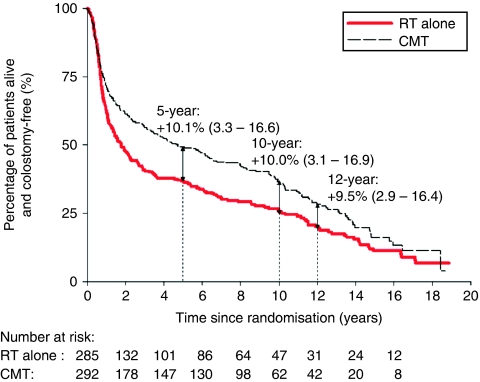
Colostomy-free survival, by treatment. Estimates shown are the absolute risk differences: combined modality therapy (CMT) minus RT alone (95% confidence interval (CI)). Median: RT alone: 1.8 years; CMT: 4.7 years. Hazard ratio (HR): 0.76 (95% CI: 0.63–0.91).

Combined modality therapy was associated with a decrease in the risk of having a colostomy or death (HR 0.76, 95% CI 0.63–0.91, *P*=0.004). The absolute risk difference remained ∼10% favouring CMT between 5 and 12 years (Appendix Table A1 and Appendix Figure A1).

### Late morbidity

There was no evidence of a treatment difference for ulcers/radionecrosis, anorectal, genitourinary or skin-related late morbidities (Appendix Table A3).

### Overall survival and deaths from anal cancer

**Figure 2:**
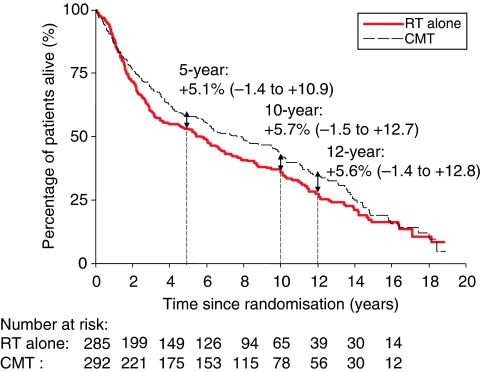
Overall survival, by treatment. The estimates shown are the absolute risk differences: combined modality therapy (CMT) minus RT alone (95% confidence interval (CI)). Median: RT alone: 5.4 years; CMT: 7.6 years. Hazard ratio (HR): 0.86 (95% CI: 0.70–1.04).

There were 405 deaths, 54% (218) from anal cancer (Table 1). The death rate was 14% lower in the CMT group, HR 0.86 (95% CI: 0.70–1.04, *P*=0.12). Five years after randomisation, the absolute risk difference of dying was 5.1% lower in the CMT group, and this remained similar up to 12 years (Appendix Table A1 and Figure 2). The median survival was 7.6 years (95% CI 5.9–9.9 years) in the CMT group and 5.4 years (95% CI 3.6–6.8 years) in those receiving RT alone.

**Figure 3:**
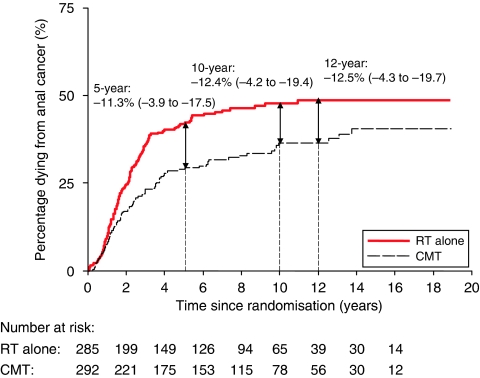
Risk of death due to anal cancer, by treatment. Estimates shown are the absolute risk differences: combined modality therapy (CMT) minus RT alone (95% confidence interval (CI)). Hazard ratio (HR): 0.67 (95% CI: 0.51–0.88).

There was a statistically significant reduction in the risk of dying from anal cancer, HR 0.67 (95% CI 0.51–0.88, *P*=0.004). Only 29 (13%) deaths from anal cancer occurred after 5 years. The absolute risk difference in death from anal cancer observed at 5 years (11.3% in favour of CMT) was similar to that observed at 12 years (12.5% Appendix Table A1 and Figure 3). Even when we classified the 10 unknown deaths as being due to anal cancer, the results did not change – HR 0.66 (95% CI: 0.50–0.85).

### Deaths other than from anal cancer

**Figure 4:**
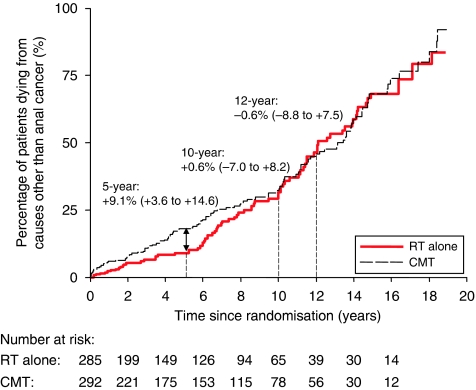
Risk of death due to causes other than anal cancer, by treatment. Estimates shown are the absolute risk differences: combined modality therapy (CMT) minus RT alone (95% confidence interval (CI)).

The assumption of proportional hazards did not hold for the first 10 years when examining deaths other than from anal cancer; Figure 4 (test for proportional hazards *P*=0.01). There were more non-anal cancer deaths in the CMT group in the first 10 years after randomisation (67 CMT *vs* 48 RT alone), with a maximum difference at about 5 years (+9.1%, 95% CI +3.6 to +14.6%, *P*=0.001). The effect almost disappeared by 10 years and remained so for the rest of the follow-up period. Appendix Table A2 provides details of the cause of death and when they occurred. During the first 5 years, many of these deaths (43 CMT *vs* 19 RT alone) were cardiovascular (15 *vs* 8 deaths, *P*=0.15), but there were smaller differences for other causes: treatment-related deaths (6 *vs* 3), and pulmonary disease (4 *vs* 0), which also contributed to the total excess risk. Four of the 23 cardiovascular deaths occurred within 40 days after the end of the initial treatment; all others occurred after 150 days (13 after 1 year). Furthermore, there were more deaths due to second malignancies in the CMT group overall (Table 1), 34 *vs* 18 (*P*=0.03), of which 8 *vs* 2 occurred in the first 5 years and 26 *vs* 16 occurred after 5 years (many were lung cancer, 17 *vs* 7).

## Discussion

The present analysis has important differences compared with the initial report in 1996. With 405 deaths compared with 236 deaths previously, and a median follow-up of 13 years instead of 3.5 years, we can provide estimates of the effect of chemoradiation at specific time points several years after the start of treatment. To our knowledge, there are no other published long-term follow-up data on chemoradiation and anal cancer from a large randomised trial. We have also shown that there is an excess of non-anal cancer deaths in the first few years after treatment, which diminishes by 10 years.

Our long-term data confirm the treatment effects we previously reported in 1996. The HRs in the earlier analysis compared with those reported here are locoregional failure 0.54 and 0.46, death from anal cancer 0.71 and 0.67, and OS 0.86 for both analyses. However, and more importantly, we further show that the full benefit can be seen by about 5 years after the start of treatment and this is sustained at least 7 years later. Twelve years after starting treatment, for every 100 patients given CMT, there are 25.3 fewer patients with a locoregional recurrence, 12.0 more who are alive and relapse free, 5.6 more who are alive and 12.5 fewer deaths from anal cancer, compared with 100 patients given RT alone. These data provide reassurance to both anal cancer patients and clinicians that CMT treatment is associated with long-term clinical benefits.

In all, 84% of recurrences are detected within the first 2 years. Although this is perhaps not surprising for squamous cell carcinoma, it has not been emphasised previously in the literature. It is difficult to be certain that tumours that relapse locally after 3 years are true recurrences, or should be regarded as second primary tumours.

Our results were statistically significant for all end points, except OS, which was probably due to the excess of deaths not from anal cancer in the CMT group in the first 5 years. However, the upper limit of the 95% CI for the OS HR was 1.04, close enough to unity to suggest that there is likely to be a beneficial effect of CMT. Most anal cancer deaths occurred in the first few years (53% in the first 2 years) as expected, and patients who survive to 5 years might be considered cured and therefore more likely to die from other causes after this time.

The ACT I data are consistent with other anal cancer trials using 5-fluorouracil and mitomycin in conjunction with radiotherapy. Five-year locoregional recurrence rates were 32% in ACT I, 33% in the EORTC study (Bartelink *et al*, 1997) and 25% in a study from the United States (Ajani *et al*, 2008). In an earlier US study, 4-year locoregional recurrence was 16% (Flam *et al*, 1996).

There was an increase in the risk of death from causes other than anal cancer in the first 5 years among patients in the CMT group, but this is relatively small when considering the large reduction in deaths from anal cancer. Cardiovascular toxicity, either during or shortly after completion of chemotherapy, was a moderate cause of death. Several chemotherapy agents have cardiotoxic effects, including 5-fluorouracil and mitomycin (Cunningham *et al*, 2008; Daher and Yeh, 2008; Viale and Yamamoto, 2008). Common complications are ischaemia, myocardial infarction, thrombosis and hypertension. Several potential mechanisms have been proposed that might account for cardiovascular toxicity, including dysfunction or damage of endothelial cells, increased platelet aggregation and modulation of nitric oxide levels (Daher and Yeh, 2008).

The difference in the occurrence of second malignancies may be partly explained by more patients in the CMT group living longer than those in the RT group, hence their cumulative risk of developing a new tumour increases with age. This is consistent with studies that show that younger age is a risk factor for the formation of second malignancies (Schwager *et al*, 2000; Tubiana, 2009). There were nearly twice as many deaths due to second malignancies in the CMT arm (34 *vs* 18 RT alone), most occurring after 5 years. About half were lung cancer (17 CMT *vs* 7 RT alone), which may reflect the shared role of smoking in the aetiology of anal cancer and lung cancer (Frisch *et al*, 1999; Daling *et al*, 2004). An excess of second malignancies has also been noted after administration of alkylating agents such as mitomycin C (Myerson *et al*, 1995), although the effect is not consistently seen across studies (Zakotnik *et al*, 2007).

There were no colostomies recorded after November 1995, and this information was not routinely collected after 2000. This is a limitation of our analysis. However, for colostomy-free survival, the time-to-event curve and event rates are similar to those for RFS. This is not surprising, as only 28% of colostomies occurred beyond 1 year after randomisation and 32% of patients had their first relapse after the first year.

Patients were generally staged clinically rather than radiologically, as were most other trials at the time (including the EORTC 22861 and RTOG 87-04 trials), as they predate the use of transrectal ultrasound and magnetic resonance imaging. Patients in the ACT I trial were likely to be understaged, owing to the lack of a surgical specimen after chemoradiation for histopathological staging, particularly in the case of microscopic involvement of non-palpable lymph nodes. The lack of radiological staging and the different TNM system in the trial (UICC 1985), based on anatomical extent, rather than size, make comparisons with modern trials difficult to interpret.

Although ACT I started more than 20 years ago, and was based on combining radiotherapy with 5-fluorouracil and mitomycin C, subsequent research conducted since then has not found chemotherapy regimens that are substantially more effective at improving local and distant control. Two large anal cancer trials examined whether cisplatin could be used instead of mitomycin. In the RTOG 98-11 trial from the United States (Ajani *et al*, 2008; *N*=682), there was no evidence of a difference in disease-free survival among those administered 5-fluorouracil plus cisplatin compared with those administered 5-fluorouracil plus mitomycin; HR 1.20, 95% CI 0.93–1.55. However, cisplatin was associated with a significantly higher colostomy rate (19 *vs* 10%, *P*=0.02). The preliminary results of the second UK Anal Cancer Trial (ACT II; James *et al*, 2009; *N*=940) suggest a 95% response rate for either mitomycin or cisplatin. Both trials concluded that cisplatin offered no additional benefit to patients, other than lower haematological toxicity. The ACT II study will have a more thorough reporting of cardiovascular toxicities and second malignancies.

In conclusion, long-term follow-up of ACT I confirms that the lower local failure rate and improvements in RFS for anal cancer following chemoradiation are maintained even 12 years after starting treatment. Only 7% (39) of patients developed metastatic disease without earlier locoregional relapse; hence the focus of treatment should concentrate on locoregional control. It is also striking that there were only 11 further locoregional relapses after 5 years. For this reason, 3-year or perhaps 5-year RFS might represent a satisfactory alternate early end point than OS, and seems to be more relevant in a population with a median age of 64 years. This finding also questions the relevance of stringent follow-up after several years have elapsed.

## Figures and Tables

**Table 1:** Type of event, by treatment

**Event**	**RT alone (*N*=285)**		**CMT (*N*=292)**	
*First event: relapse or death*				
Locoregional relapse^a^	155		94	
Locoregional relapse and distant relapse^a^	7		7	
Distant relapse	21		29	
Death	56		83	
Any event	239		213	
				
*Cause of death*				
Anal cancer	125		93	
New cancer	18^b^		34^c^	
Treatment related	5		6	
Other causes	50		64	
Cardiac		18		20
Cerebrovascular accident		5		6
Other vascular		3		3
Infection		15		17
Pulmonary		5		9
Other		4		9
Unknown	7		3	
Total	205		200	

Abbreviations: CMT=combined modality therapy; RT=radiotherapy.

a Within 6 weeks following the end of initial treatment, there were two RT-alone patients and seven CMT patients who died, and nine RT-alone patients and ten CMT patients who had a relapse.

b Lung (7), unknown primary (4), ureter (1), oesophagus (1), liver (1), laryngeal (1), gastric (1), large bowel (1), unknown (1).

c Lung (17), leukaemia (3), breast (2), rectal (1), prostate (1), bladder (1), cerebral glioma (1), neuroendocrine (1), colon (1), myelodysplasia (1), ovary (1), oesophagus (1), larynx (1), abdomen (1), unknown primary (1).

**Table A1:** Summary results on efficacy

**Event**	**RT alone**	**CMT**
*Locoregional relapse rate (%) at*		
3 years	53.4	29.7
5 years	57.1	32.3
10 years	59.1	33.8
12 years	59.1	33.8
		
*Relapse-free survival rate (%) at*		
3 years	38.4	51.1
5 years	33.7	46.6
10 years	23.5	36.2
12 years	17.7	29.7
		
*Colostomy-free survival rate (%) at*		
3 years	40.5	50.4
5 years	36.8	46.9
10 years	25.6	35.6
12 years	20.1	29.6
		
*Overall survival rate (%) at*		
3 years	60.0	64.6
5 years	53.0	58.1
10 years	35.8	41.5
12 years	27.5	33.1
		
*Anal cancer death rate (%) at*		
3 years	35.9	25.9
5 years	41.8	30.5
10 years	47.7	35.3
12 years	48.7	36.2

Abbreviations: CMT=combined modality therapy; RT=radiotherapy.

**Table A2:** Type of event, by time to event and treatment

	**0–1.9 years**				**2.0–4.9 years**				**5.0–9.9 years**				**10+ years**			
**Event**	**RT alone (*N*=285)**		**CMT (*N*=292)**		**RT alone**		**CMT**		**RT alone**		**CMT**		**RT alone**		**CMT**	
*First event: relapse or death*																
Locoregional relapse	136		79		14		9		4		5		1		1	
Distant relapse	16		19		4		5		1		3		0		2	
Locoregional relapse and distant relapse	6		6		1		1		0		0		0		0	
Death	5		11		6		17		20		24		25		31	
Any event	163		115		25		32		25		32		26		34	
																
*Cause of death*																
Anal cancer	68		47		44		30		12		13		1		3	
New cancer	0		3		2		5		9		9		7		17	
Treatment related	3		5		0		1		1		0		1		0	
Other causes	7		14		4		14		17		15		22		21	
Cardiac		1		3		2		5		7		5		8		7
Cerebrovascular accident		2		3		0		2		1		0		2		1
Other vascular		2		2		1		0		0		1		0		0
Infection		0		2		1		1		5		6		9		8
Pulmonary		0		1		0		3		3		2		2		3
Other		2		3		0		3		1		1		1		2
Unknown	3		0		0		1		2		0		2		2	
Total	81		69		50		51		41		37		33		43	

Abbreviations: CMT=combined modality therapy; RT=radiotherapy.

**Table A3:** Late morbidities

	**% (*n*)**		
**Late morbidity type**	**RT alone (*N*=281)**	**CMT (*N*=284)**	***P*-value**
Ulcers/radionecrosis	6 (18)	8 (23)	0.44
Anorectal	27 (76)	29 (81)	0.70
Genitourinary	4 (11)	4 (11)	0.98
Skin	18 (51)	21 (59)	0.43

Abbreviations: CMT=combined modality therapy; RT=radiotherapy.

## Appendix

Members of the original Working Party: Sidney Arnott, David Cunningham, Jill Gallagher, Richard Gray, Jack Hardcastle, Joan Houghton, Roger James, Tina Lennon, Helen Meadows, Jean Mossman, John Northover, David Morgan, Nicholas Plowman and Maurice Slevin. Figure A1, Table A1, Table A2 and Table A3.
